# Non-Markovian Diffusion and Adsorption–Desorption Dynamics: Analytical and Numerical Results

**DOI:** 10.3390/e26040294

**Published:** 2024-03-27

**Authors:** Derik W. Gryczak, Ervin K. Lenzi, Michely P. Rosseto, Luiz R. Evangelista, Rafael S. Zola

**Affiliations:** 1 Independent Researcher, Irati 84507-012, PR, Brazil; derikwilliam.gryczak@gmail.com; 2 Departamento de Física, Universidade Estadual de Ponta Grossa, Ponta Grossa 84030-900, PR, Brazil; michelyrosseto@gmail.com; 3 Departamento de Física, Universidade Estadual de Maringá, Maringá 87020-900, PR, Brazil; lre@dfi.uem.br; 4 Istituto dei Sistemi Complessi (ISC–CNR), Via dei Taurini, 19, 00185 Rome, Italy; 5 Department of Molecular Science and Nanosystems, Ca’ Foscari University of Venice, Via Torino 155, 30175 Mestre (VE), Italy; 6 Department of Physics, Universidade Tecnológica Federal do Paraná, Apucarana 86812-460, PR, Brazil; rzola1@kent.edu

**Keywords:** anomalous diffusion, adsorption–desorption, surface effects

## Abstract

The interplay of diffusion with phenomena like stochastic adsorption–desorption, absorption, and reaction–diffusion is essential for life and manifests in diverse natural contexts. Many factors must be considered, including geometry, dimensionality, and the interplay of diffusion across bulk and surfaces. To address this complexity, we investigate the diffusion process in heterogeneous media, focusing on non-Markovian diffusion. This process is limited by a surface interaction with the bulk, described by a specific boundary condition relevant to systems such as living cells and biomaterials. The surface can adsorb and desorb particles, and the adsorbed particles may undergo lateral diffusion before returning to the bulk. Different behaviors of the system are identified through analytical and numerical approaches.

## 1. Introduction

Describing the physical world is complex and necessitates employing various methods, both experimental and theoretical, to grasp and interpret the behavior of systems. Diffusion is prevalent in various situations that can be usual or anomalous and may appear combined with different processes, such as adsorption–desorption [1] and reaction–diffusion [2]. In the case of normal diffusion, the system exhibits Markovian characteristics [3], particularly the mean square displacement with a linear dependence on time, i.e., $\langle {\left(r-\langle r\rangle \right)}^{2}\rangle ∼t$. In contrast, anomalous diffusion results in stochastic processes that govern the system exhibiting non-Markovian features [4], yielding a nonlinear relationship for the mean square displacement, e.g., $\langle {\left(r-\langle r\rangle \right)}^{2}\rangle ∼t^{\alpha }$, where α<1 or α>1 corresponds to sub- or superdiffusion, respectively [5,6]. In conjunction with these situations, adsorption–desorption or reaction processes may occur, directly impacting various aspects of the system. These phenomena play a relevant role in many scenarios, such as antibody binding and coupling to a receptor in a cell [7], electrical impedance [5,8], polymer dynamics at solid–liquid interfaces [9], molecule traveling through a cellular membrane [10], the dynamics of loci in a chromosome [11], the movement of a tracer particle [12], catalytic kinetics [13,14], and, in particular, in living systems [15,16,17]. The particles (or molecules) adsorbed by the surface can diffuse or become immobile and, after some time, can be desorbed to the bulk. In this manner, the system can also present lateral diffusion associated with the adsorbed particles. This behavior has been observed in different systems; for example, lateral diffusion occurs in a cellular membrane by lipids and proteins, which may occur in different modes, such as homogeneous or hop diffusion [18,19], and, in addition, on a tubular membrane, which plays a vital role in neuronal axons by transporting signaling molecules and proteins [20]. Lateral diffusion is not restricted to biological systems and is also found in graphene oxide sheets dispersed in solvents such as water [21], and charge carriers find their place in semiconductors through the lateral photoelectric effect [22].

This study investigates a heterogeneous diffusion process for a three-dimensional system subjected to an adsorption–desorption process on a surface. The diffusion equation governs the bulk particles with spatial dependence on the diffusion coefficient in the *z*-direction. The adsorption–desorption process occurs on the surface located at the point z=0. The surface is made up of the plane (x,y), and the kinetic terms explain the interaction with the particles in the bulk. These terms represent the adsorption–desorption process of the particles from the bulk to the surface (adsorption) and from the surface to the bulk (desorption). Adsorbed particles can diffuse on the surface and, after some time, are desorbed to the bulk. However, some particles will be desorbed in the next step since the process is stochastic. We consider that the particles can diffuse on the surface prior to desorption. We only consider inhomogeneity in the bulk, represented by a spatial dependence on the diffusion coefficient in the *z*-direction. These features imply a coupling between the processes in the bulk and on the surface, where each process influences the other. This analysis explores the relationship between bulk processes and surface processes, particularly examining the role of bulk heterogeneity in regulating surface behavior. This is performed in Section 2, where the analytical and numerical calculations are presented and discussed. In Section 3, we discuss the results and offer some conclusions about the behavior exhibited in the problems analyzed here.

## 2. The Problem: Diffusion and Kinetics

Let us initiate our analysis by considering that the subsequent equation controls the diffusion process in the bulk:

(1) $$\begin{array}{c} \frac{\partial }{\partial t}\rho_{\mathrm{bulk}}\left(r,t\right)=D_{b,\|}^{\;}\nabla_{\|}^{2}\rho_{\mathrm{bulk}}\left(r,t\right)+\nabla_{⊥}\cdot \left[D_{b,⊥}\left(r\right)\nabla_{⊥}\rho_{\mathrm{bulk}}\left(r,t\right)\right]. \end{array}$$

Equation (1) is the typical continuity equation applied to Fick’s law, i.e.,

(2) $$\begin{array}{c} \frac{\partial }{\partial t}\rho_{\mathrm{bulk}}\left(r,t\right)+\nabla \cdot J_{\mathrm{bulk}}\left(r,t\right)=0 \end{array}$$

combined with $J_{\mathrm{bulk}}\left(r,t\right)$=$-D_{b,\|}\nabla_{\|}\rho_{\mathrm{bulk}}\left(r,t\right)-D_{b,⊥}\left(r\right)\nabla_{⊥}\rho_{\mathrm{bulk}}\left(r,t\right)$, which states that the time variation in the density of particles in the bulk is equal to the spatial change caused by concentration gradients. We assume that the spatial term is composed by a diffusion process taking place in the bulk, where there is heterogeneity in the $\hat{z}$ direction, but particles can diffuse in 3D. More specifically, $D_{b,⊥}\left(r\right)=D_{b,⊥}r_{⊥}^{-\eta }$ (−1≤η gives the degree of heterogeneity for the *z* direction), $\nabla_{⊥}\cdot \left[r_{⊥}^{-\eta }\nabla_{⊥}\rho \left(r,t\right)\right]=\partial_{z}[{\left|z\right|}^{-\eta }\partial_{z}\rho \left(r,t\right)]$, and $\nabla_{\|}^{2}=\partial_{x}^{2}+\partial_{y}^{2}$, where $r_{\|}=x\hat{x}+y\hat{y}$ and $r_{⊥}=z\hat{z}$. $D_{b,\|}$ and $D_{b,⊥}$ are the diffusion coefficients in the parallel and perpendicular directions, and $\rho_{\mathrm{bulk}}\left(r,t\right)$ represents the distribution of particles in bulk, in units of particles per volume. Note that the diffusion coefficients $D_{b,||}$ and $D_{b,⊥}$ are connected to diffusion in the plane (x,y) and in the perpendicular direction *z*, respectively. We should mention that the form of the diffusion coefficient poses a scale-dependent dispersivity for diffusion in the $\hat{z}$ direction and thus creates heterogeneity in the bulk. These forms of the diffusion coefficients allow us to consider anisotropic diffusion and enable us to analyze scenarios related to anomalous diffusion, where the mean square displacement has a nonlinear time dependence. We underline that similar spatial dependence on the diffusion coefficient has successfully been used to investigate diffusion on fractals [23,24,25], turbulence [26], solute transport in fractal porous media [27,28], and atom deposition in a porous substrate [29]. For the isotropic situations, we have $D_{b,||}=D_{b,⊥}$ in the bulk.

For the processes that occur on the surface, we assume that the bulk particles can be adsorbed by the surface located in z=0. Once adsorbed, particles can diffuse within the surface and a reaction process may take place; this is intimately related to several technologies, such as catalysts for the production of biochemical sensors [30], fuel [31], energy devices [32], and many others. Hence, the following equation is considered:

(3) $$\begin{array}{c} \begin{array}{cc} \frac{\partial }{\partial t}\rho_{\mathrm{surf}}\left(r_{\|},t\right) & =D_{s}\nabla_{\|}^{2}\rho_{\mathrm{surf}}\left(r_{\|},t\right)+\int_{0}^{t}dt^{\prime }k_{\mathrm{ads}}\left(t-t^{\prime }\right)\rho_{\mathrm{bulk}}\left(r,t^{\prime }\right){|}_{z=0}\\ & -\int_{0}^{t}dt^{\prime }k_{\mathrm{total}}\left(t-t^{\prime }\right)\rho_{\mathrm{surf}}\left(r_{\|},t\right), \end{array} \end{array}$$

where $D_{s}$ is the diffusion coefficient for the particles’ concentration (or distribution) on the surface, $\rho_{\mathrm{surf}}\left(r_{\|},t\right)$ represents the density of particles on the surface (particles per area), $k_{\mathrm{total}}\left(t\right)$ is a kernel that can be connected to the desorption and reaction processes, i.e., $k_{\mathrm{total}}\left(t\right)=k_{\mathrm{surf},r}\left(t\right)+k_{\mathrm{desor}}\left(t\right)$, which may be present on the surface. $k_{\mathrm{desor}}\left(t\right)$ is related to the desorption rate, and $k_{\mathrm{surf},r}\left(t\right)$ is related to the reaction processes on the surfaces during the diffusion process on the surface. Equation (3) can be obtained by considering the continuity with additional terms related to the interaction between the surface and bulk, i.e.,

(4) $$\begin{array}{c} \begin{array}{cc} \frac{\partial }{\partial t}\rho_{\mathrm{surf}}\left(r_{\|},t\right) & +\nabla_{\|}\cdot J_{\mathrm{surf}}\left(r_{\|},t\right)=\int_{0}^{t}dt^{\prime }k_{\mathrm{ads}}\left(t-t^{\prime }\right)\rho_{\mathrm{bulk}}\left(r,t^{\prime }\right){|}_{z=0}\\ & -\int_{0}^{t}dt^{\prime }k_{\mathrm{total}}\left(t-t^{\prime }\right)\rho_{\mathrm{surf}}\left(r_{\|},t^{\prime }\right), \end{array} \end{array}$$

combined with Fick’s law, i.e., $J_{\mathrm{surf}}\left(r_{\|},t\right)=-D_{s}\nabla_{\|}\rho_{\mathrm{surf}}\left(r_{\|},t\right)$. In this manner, Equation (3) (or Equation (4)) has terms that represent an interaction between the surface and the particles after the adsorption processes, where they can be desorbed or promote the formation of other particles. In the latter case, the kernel may also be connected to the presence of intermediate processes during the reaction. The kernel $k_{\mathrm{ads}}\left(t\right)$ governs the adsorption process, i.e., it tells us what the interaction is between the surface particles and the bulk ones. Thus, depending on the nature of this interaction, the adsorption–desorption phenomena may or may not follow nonexponential decay behavior. For example, when we consider pure physisorption processes, the subsequent state of a particle depends on its preceding state, which is a way to characterize a non-Markovian process, i.e., a process involving a memory effect [33]. For $k_{\mathrm{ads}}\left(t\right)\propto \delta \left(t\right)$, the kernel is a localized function of time, implying that the preceding state does not matter to the actual state. These important features show that the kernel related to adsorption–desorption may account for a large class of effects that can be short- or long-ranged, depending on the processes occurring on the surface.

Equations (1) and (3) are coupled by the following condition

(5) $$\begin{array}{c} {\left.n\cdot \left[D_{b,⊥}r_{⊥}^{-\eta }\nabla_{⊥}\rho_{\mathrm{bulk}}\left(r,t\right)\right]\right|}_{z=0}=\frac{d}{dt}\rho_{\mathrm{surf}}\left(r_{\|},t\right)+\int_{0}^{t}dt^{\prime }k_{r}\left(t-t^{\prime }\right){\left.\rho_{\mathrm{bulk}}\left(r,t\right)\right|}_{z=0}. \end{array}$$

In Equation (5), the adsorption–desorption processes are represented by the first term on the right-hand side; it couples the processes present on the surface with the ones in the bulk. The other term, i.e., the second term, represents a reaction process (see, for example, Refs. [34,35]), where the particles are removed from the bulk to the surface. The additional boundary conditions to analyze the problem defined above are

$$\begin{array}{ccc} & & {\left.\partial_{z}\rho_{\mathrm{bulk}}\left(r,t\right)\right|}_{z\to \infty }=0,\;{\left.\partial_{x}\rho_{\mathrm{bulk}}\left(r,t\right)\right|}_{x\to \pm \infty }=0,\;{\left.\partial_{y}\rho_{\mathrm{bulk}}\left(r,t\right)\right|}_{y\to \pm \infty }=0,\\ & & {\left.\partial_{x}\rho_{\mathrm{surf}}\left(r_{\left|\right|},t\right)\right|}_{x\to \pm \infty }=0,\;\mathrm{and}\;{\left.\partial_{y}\rho_{\mathrm{surf}}\left(r_{\left|\right|},t\right)\right|}_{y\to \pm \infty }=0. \end{array}$$

For the initial condition, we consider $\rho_{\mathrm{bulk}}\left(r,0\right)=\varphi_{\mathrm{bulk}}\left(r\right)$ (so any initial configuration is allowed in the bulk) and, for simplicity, $\rho_{\mathrm{surf}}\left(r_{\left|\right|},0\right)=0$, which implies that, initially, there is no concentration of particles on the surfaces.

Performing some calculations, we can show that the processes on the surface modify the bulk, i.e.,

(6) $$\begin{array}{c} \frac{d}{dt}\left[\int dr_{⊥}\int dr_{\|}\rho_{\mathrm{bulk}}\left(r,t\right)+\int dr_{\|}\rho_{\mathrm{surf}}\left(r_{\|},t\right)\right]=-\int_{0}^{t}dt^{\prime }k_{r}\left(t-t^{\prime }\right){\left.\rho_{\mathrm{bulk}}\left(r,t\right)\right|}_{z=0}\;, \end{array}$$

where $\int dr_{⊥}\equiv \int_{0}^{\infty }dz$, $\int dr_{\|}\equiv \int_{-\infty }^{\infty }dx\int_{-\infty }^{\infty }dy$, and, consequently, for $k_{r}\left(t\right)=0$, we have

(7) $$\begin{array}{c} \int dr_{⊥}\;\;\;\int dr_{\|}\;\rho_{\mathrm{bulk}}\left(r,t\right)+\int dr_{\|}\;\rho_{\mathrm{surf}}\left(r_{\|},t\right)=\mathrm{constant}, \end{array}$$

which is a direct consequence of conserving the total number of particles in the system.

We now investigate the spatial and time behavior of the density of particles on the surface and in the bulk from analytical and numerical points of view. Thus, we start by obtaining the analytical solution for this problem, that is, closed expressions for $\rho_{\mathrm{bulk}}\left(r,t\right)$ and $\rho_{\mathrm{surf}}\left(r_{\|},t\right)$. To do this, we use integral transforms and the Green function approach [36,37]. One of the integral transforms is the Laplace transform, defined as

(8) $$L\left\{\rho_{\mathrm{bulk}}\left(r,t\right);s\right\}=\int_{0}^{\infty }dt\;e^{-st}\rho_{\mathrm{bulk}}\left(r,t\right)={\hat{\rho }}_{\mathrm{bulk}}\left(r,s\right),$$

and its inverse,

(9) $$L^{-1}\left\{{\hat{\rho }}_{\mathrm{bulk}}\left(r,s\right);t\right\}=\frac{1}{2\pi i}\int_{-i\infty +c}^{i\infty +c}ds\;e^{st}{\hat{\rho }}_{\mathrm{bulk}}\left(r,s\right)=\rho_{\mathrm{bulk}}\left(r,t\right).$$

In addition, we use the special integral transform that can be constructed from the eigenfunctions of the Sturm–Liouville problem related to the following differential equation:

(10) $$\frac{\partial }{\partial z}\left\{z^{-\eta }\frac{\partial }{\partial z}\psi \left(z,k_{z}\right)\right\}=-k_{z}^{2+\eta }\psi \left(z,k_{z}\right)\;,$$

subjected to the boundary condition $|\psi (\infty ,k_{z})|<\infty$. The eigenfunctions obtained from Equation (10) are given by

(11) $$\psi \left(z,k_{z}\right)={\left(z\;k_{z}\right)}^{\frac{1}{2}\left(1+\eta \right)}J_{-\nu }\left[\frac{2}{2+\eta }{\left(k_{z}\;z\right)}^{\frac{1}{2}\left(2+\eta \right)}\right]$$

where $J_{\nu }\left(x\right)$ denotes the Bessel function [37] with order ν=(1+η)/(2+η). Equation (11) allows us to define the following integral transform:

(12) $$\begin{array}{ccc} F_{\eta }\left\{\rho_{\mathrm{bulk}}\left(r,t\right);z\right\} & = & \int_{0}^{\infty }dz\;\psi \left(z,k_{z}\right)\rho_{\mathrm{bulk}}\left(r,t\right)={\tilde{\rho }}_{\mathrm{bulk}}\left(r_{\|},k_{z},t\right)\;, \end{array}$$

(13) $$\begin{array}{ccc} F_{\eta }^{-1}\left\{{\tilde{\rho }}_{\mathrm{bulk}}\left(r_{\|},k_{z},t\right);k_{z}\right\} & = & \int_{0}^{\infty }dk_{z}\;\psi \left(z,k_{z}\right){\tilde{\rho }}_{\mathrm{bulk}}\left(r_{\|},k_{z},t\right)=\rho_{\mathrm{bulk}}\left(r,t\right)\;. \end{array}$$

We observe that Equations (12) and (13) may be related to a generalized Hankel transform [38,39,40,41]. By using these integral transforms and Fourier transform

(14) $$F_{x,y}\left\{\rho_{\mathrm{bulk}}\left(r,t\right);x,y\right\}=\int_{-\infty }^{\infty }dxe^{-ik_{x}x}\int_{-\infty }^{\infty }dye^{-ik_{y}y}\rho_{\mathrm{bulk}}\left(r,t\right)={\bar{\rho }}_{\mathrm{bulk}}\left(k_{\|},z,t\right)$$

and it inverse

(15) $$F_{x,y}^{-1}\left\{{\bar{\rho }}_{\mathrm{bulk}}\left(k_{\|},z,t\right);k_{x},k_{y}\right\}=\frac{1}{2\pi }\int_{-\infty }^{\infty }dk_{x}e^{ik_{x}x}\frac{1}{2\pi }\int_{-\infty }^{\infty }dk_{y}\;e^{ik_{y}y}{\bar{\rho }}_{\mathrm{bulk}}\left(k_{\|},z,t\right)=\rho_{\mathrm{bulk}}\left(r,t\right),$$

where $k_{\|}=\left(k_{x},k_{y}\right)$, we have

(16) $$\begin{array}{c} \begin{array}{cc} {\hat{\bar{\rho }}}_{\mathrm{bulk}}\left(k_{\|},z,s\right) & =-\int_{0}^{\infty }dz^{\prime }{\tilde{\varphi }}_{\mathrm{bulk}}\left(k_{\|},z^{\prime }\right)\hat{\bar{G}}\left(k_{\|},z,z^{\prime },s\right)\\ & +\left[s{\hat{\bar{\rho }}}_{\mathrm{surf}}\left(k_{\|},s\right)-{\hat{k}}_{r}\left(s\right){\hat{\bar{\rho }}}_{\mathrm{bulk}}\left(k_{\|},0,s\right)\right]\hat{\bar{G}}\left(k_{\|},0,z,s\right), \end{array} \end{array}$$

and

(17) $$\begin{array}{c} {\hat{\bar{\rho }}}_{\mathrm{surf}}\left(k_{\|},s\right)=\frac{{\hat{k}}_{\mathrm{ads}}\left(s\right)}{s+D_{s}{\left|k_{\|}\right|}^{2}+{\hat{k}}_{\mathrm{total}}\left(s\right)}{\hat{\bar{\rho }}}_{\mathrm{bulk}}\left(k_{\|},0,s\right), \end{array}$$

for the initial condition previously defined, which assumes that the particles are initially in bulk. The Green function is given by

(18) $$\begin{array}{c} \bar{G}\left(k_{\|},z,z^{\prime },t\right)=-\frac{e^{-D_{b,\|}{\left|k_{\|}\right|}^{2}t}}{\left(2+\eta \right)D_{b,⊥}t}{\left(zz^{\prime }\right)}^{\frac{1}{2}\left(1+\eta \right)}e^{-\frac{1}{{\left(2+\eta \right)}^{2}D_{b,⊥}t}\left(z^{2+\eta }+z^{\prime 2+\eta }\right)}I_{-\nu }\left[\frac{2{\left(zz^{\prime }\right)}^{\frac{1}{2}\left(2+\eta \right)}}{{\left(2+\eta \right)}^{2}D_{b,⊥}t}\right]\;, \end{array}$$

where $I_{-\nu }\left(x\right)$ is the Bessel function of the modified argument [37]. From the inverse of the Fourier transform, we obtain the Green function in the form

(19) $$\begin{array}{c} \begin{array}{cc} G(r_{\|},z,z^{\prime },t) & =-\frac{1}{4\pi D_{b,\|}t}e^{\frac{|r_{\|}{|}^{2}}{4D_{b,\|}t}}\\ & \times \frac{{\left(zz^{\prime }\right)}^{\frac{1}{2}\left(1+\eta \right)}}{\left(2+\eta \right)D_{b,⊥}t}e^{-\frac{1}{{\left(2+\eta \right)}^{2}D_{b,⊥}t}\left(z^{2+\eta }+z^{\prime 2+\eta }\right)}I_{-\nu }\left[\frac{2{\left(zz^{\prime }\right)}^{\frac{1}{2}\left(2+\eta \right)}}{{\left(2+\eta \right)}^{2}D_{b,⊥}t}\right]\;. \end{array} \end{array}$$

**Figure 1:**
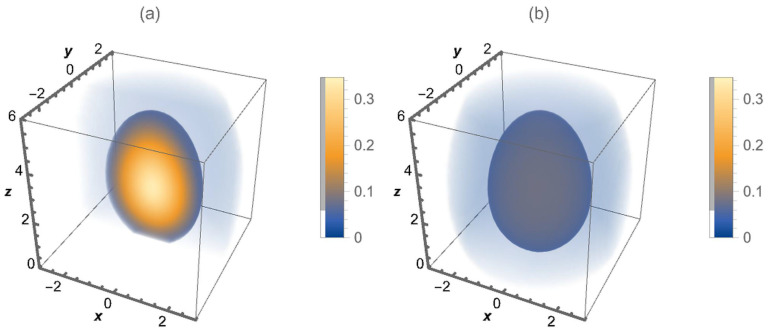
A three-dimensional density plot for the Green function (Equation (19)) when η=−0.5. In (**a**) a cut is represented through the center in the y direction, while (**b**) shows the behavior of the Green function around the point r=(0,0,1), i.e., how the particles are distributed. For simplicity, we consider $D_{b,\|}t=D_{b,⊥}t=0.5$ and $z^{\prime }=1.0$, in arbitrary units.

**Figure 2:**
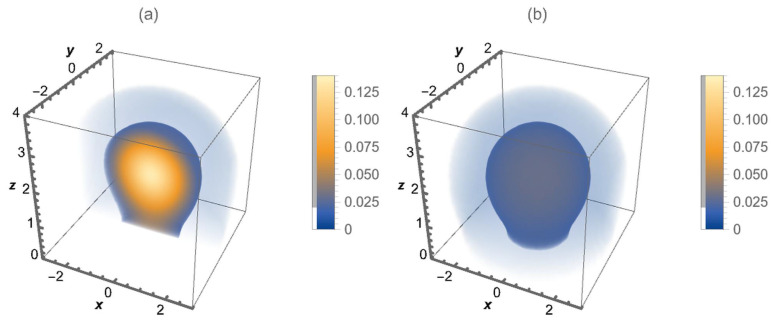
A three-dimensional density plot for the Green function (Equation (19)) when η=0.5. In (**a**) a cut is represented through the center in the y direction, while (**b**) shows the behavior of the Green function around the point r=(0,0,1), i.e., how the particles are distributed. For simplicity, we consider $D_{b,\|}t=D_{b,⊥}t=0.5$ and $z^{\prime }=1.0$, in arbitrary units.

It is illustrative to look at the behavior of the Green function (Equation (19)) exhibited in Figure 1a,b. These are depicted for η=−0.5, while Figure 2a,b illustrate the behavior of the Green function for η=0.5. The spatial dependence on the diffusion coefficient obtained for different values of η is responsible for different behaviors of the Green function and, consequently, for the spread of the distributions when the system is characterized by this type of heterogeneity.

Before proceeding, we underline that Equation (18) is obtained by solving the following equation

(20) $$\begin{array}{c} \begin{array}{cc} D_{b,⊥}\frac{\partial }{\partial z}\left\{z^{-\eta }\frac{\partial }{\partial z}\bar{G}\left(k_{\|},z,z^{\prime },t\right)\right\}\;\;\; & -\;\;\;D_{b,\|}{\left|k_{\|}\right|}^{2}\bar{G}\left(k_{\|},z,z^{\prime },t\right)\\ \;\;\; & -\;\;\;\frac{\partial }{\partial t}\bar{G}\left(k_{\|},z,z^{\prime },t\right)=\delta \left(z-z^{\prime }\right)\delta \left(t\right)\;, \end{array} \end{array}$$

subjected to the boundary conditions

(21) $$\begin{array}{c} {\left.D_{b,⊥}z^{-\eta }\frac{\partial }{\partial z}\bar{G}\left(k_{\|},z,z^{\prime },t\right)\right|}_{z=0}=0\;\;,\;\;{\left.D_{b,⊥}z^{-\eta }\frac{\partial }{\partial z}\bar{G}\left(k_{\|},z,z^{\prime },t\right)\right|}_{z=\infty }=0, \end{array}$$

taking into account that $\bar{G}\left(k_{\|},z,z^{\prime },t\right)=0$ for t<0.

Using these results, it is possible to obtain the profile of the density of particles in the bulk and on the surface. In particular, before the inversion procedures, we may notice that

(22) $$\begin{array}{c} \begin{array}{cc} {\hat{\bar{\rho }}}_{\mathrm{surf}}\left(k_{\|},s\right) & =-\frac{{\hat{k}}_{\mathrm{ads}}\left(s\right)}{(s+D_{s}|k_{\|}{|}^{2}+{\hat{k}}_{\mathrm{total}}\left(s\right))\left(1-k_{r}\left(s\right)\hat{\bar{G}}\left(k_{\|},0,0,s\right)\right)-s{\hat{k}}_{\mathrm{ads}}\left(s\right)\hat{\bar{G}}\left(k_{\|},0,0,s\right)}\\ & \times \int_{0}^{\infty }dz^{\prime }{\bar{\varphi }}_{\mathrm{bulk}}\left(k_{\|},z^{\prime }\right)\hat{\bar{G}}\left(k_{\|},0,z^{\prime },s\right)\;, \end{array} \end{array}$$

and

(23) $$\begin{array}{c} \begin{array}{cc} {\hat{\bar{\rho }}}_{\mathrm{bulk}}\left(k_{\|},z,s\right) & =-\int_{0}^{\infty }dz^{\prime }{\tilde{\varphi }}_{\mathrm{bulk}}\left(k_{\|},z^{\prime }\right)\hat{\bar{G}}\left(k_{\|},z,z^{\prime },s\right)\\ & -\frac{(s+D_{s}|k_{\|}{|}^{2}+{\hat{k}}_{\mathrm{total}}\left(s\right))\hat{\bar{G}}\left(k_{\|},0,z,s\right)}{(s+D_{s}|k_{\|}{|}^{2}+{\hat{k}}_{\mathrm{total}}\left(s\right))\left(1-k_{r}\left(s\right)\hat{\bar{G}}\left(k_{\|},0,0,s\right)\right)-s{\hat{k}}_{\mathrm{ads}}\left(s\right)\hat{\bar{G}}\left(k_{\|},0,0,s\right)}\\ & \times \left[\frac{sk_{\mathrm{ads}}\left(s\right)}{s+D_{s}{\left|k_{\|}\right|}^{2}+{\hat{k}}_{\mathrm{total}}\left(s\right)}+k_{r}\left(s\right)\right]\int_{0}^{\infty }dz^{\prime }{\bar{\varphi }}_{\mathrm{bulk}}\left(k_{\|},z^{\prime }\right)\hat{\bar{G}}\left(k_{\|},0,z^{\prime },s\right)\;. \end{array} \end{array}$$

At this point, we have the tools in the Laplace–Fourier space that are needed to consider the following two particular cases of the previous equations.

The first refers to the absorption process of particles by the surface in the absence of reaction processes, that is, when ${\hat{k}}_{\mathrm{total}}\left(s\right)=0$ and ${\hat{k}}_{r}\left(s\right)=0$, at a constant absorption rate, that is, ${\hat{k}}_{\mathrm{ads}}\left(s\right)=k_{\mathrm{ads}}$, with the particles initially in bulk. In this case, Equations (22) and (23) are reduced, respectively, to

(24) $$\begin{array}{c} {\hat{\bar{\rho }}}_{\mathrm{surf}}\left(k_{\|},s\right)=-\frac{k_{\mathrm{ads}}}{s+D_{s}{\left|k_{\|}\right|}^{2}-sk_{\mathrm{ads}}\hat{\bar{G}}\left(k_{\|},0,0,s\right)}\int_{0}^{\infty }dz^{\prime }{\bar{\varphi }}_{\mathrm{bulk}}\left(k_{\|},z^{\prime }\right)\hat{\bar{G}}\left(k_{\|},0,z^{\prime },s\right) \end{array}$$

and

(25) $$\begin{array}{c} \begin{array}{cc} {\hat{\bar{\rho }}}_{\mathrm{bulk}}\left(k_{\|},z,s\right) & =-\int_{0}^{\infty }dz^{\prime }{\tilde{\varphi }}_{\mathrm{bulk}}\left(k_{\|},z^{\prime }\right)\hat{\bar{G}}\left(k_{\|},z,z^{\prime },s\right)\\ & -\frac{sk_{\mathrm{ads}}\hat{\bar{G}}\left(k_{\|},0,z,s\right)}{s+D_{s}{\left|k_{\|}\right|}^{2}-sk_{\mathrm{ads}}\hat{\bar{G}}\left(k_{\|},0,0,s\right)}\int_{0}^{\infty }dz^{\prime }{\bar{\varphi }}_{\mathrm{bulk}}\left(k_{\|},z^{\prime }\right)\hat{\bar{G}}\left(k_{\|},0,z^{\prime },s\right)\;. \end{array} \end{array}$$

After obtaining the inverses of the Laplace and Fourier transforms of both equations, we arrive at the final expressions for the density of particles on the surface and in the bulk, namely,

(26) $$\begin{array}{c} \begin{array}{cc} \rho_{\mathrm{surf}}\left(r_{\|},s\right) & =-k_{\mathrm{ads}}\int dr_{\|}^{\prime }\int_{0}^{t}dt^{\prime }G^{\left(1\right)}\left(r_{\|}-r_{\|}^{\prime },t-t^{\prime }\right)\\ & \times \int dr_{\|}^{\prime \prime }\int_{0}^{\infty }dz^{\prime }\varphi_{\mathrm{bulk}}\left(r_{\|}^{\prime }-r_{\|}^{\prime \prime },z^{\prime }\right)G\left(r_{\|},0,z^{\prime },t^{\prime }\right) \end{array} \end{array}$$

with

(27) $$\begin{array}{c} \begin{array}{cc} G^{\left(1\right)}\left(r_{\|},t\right) & =\sum_{m=0}^{n}\left(\begin{array}{c} n\\ m \end{array}\right)\int_{0}^{t}dt^{\prime }\frac{{\left(t-t^{\prime }\right)}^{\frac{1+\eta }{2+\eta }n-1}t^{\prime m}}{\Gamma \left(\frac{1+\eta }{2+\eta }n\right)\Gamma \left(1+m\right)}\\ & \times \int r_{\|}^{\prime }G_{D_{s},xy}\left(r_{\|}^{\prime },t-t^{\prime }\right)\frac{\partial^{m}}{\partial t^{\prime m}}G_{D_{b,\|},xy}\left(r_{\|}-r_{\|}^{\prime },t^{\prime }\right), \end{array} \end{array}$$

where

(28) $$\begin{array}{c} G_{D_{i},xy}\left(r_{\|},t\right)=\frac{1}{4\pi D_{i}t}\exp\left(-\frac{r_{\|}^{2}}{4D_{i}t}\right)\;, \end{array}$$

and

(29) $$\begin{array}{ccc} \rho_{\mathrm{bulk}}\left(r_{\|},z,s\right) & = & -\int dr_{\|}^{\prime }\int_{0}^{\infty }dz^{\prime }\varphi_{\mathrm{bulk}}\left(r_{\|}^{\prime },z^{\prime }\right)G\left(r_{\|}-r_{\|}^{\prime },z,z^{\prime },t\right)\\ & - & k_{\mathrm{ads}}\int dr_{\|}^{\prime }\int_{0}^{t}dt^{\prime }G^{\left(1\right)}\left(r_{\|}-r_{\|}^{\prime },t-t^{\prime }\right)\int_{0}^{t^{\prime }}dt^{\prime \prime }\int dr_{\|}^{\prime \prime }G\left(r_{\|}^{\prime }-r_{\|}^{\prime \prime },0,z,t^{\prime }-t^{\prime \prime }\right)\\ & \times & \int dr_{\|}^{\prime \prime \prime }\int_{0}^{\infty }dz^{\prime }\varphi_{\mathrm{bulk}}\left(r_{\|}^{\prime \prime }-r_{\|}^{\prime \prime \prime },z^{\prime }\right)G\left(r_{\|}^{\prime \prime \prime },0,z^{\prime },t^{\prime \prime }\right)\;. \end{array}$$

**Figure 3:**
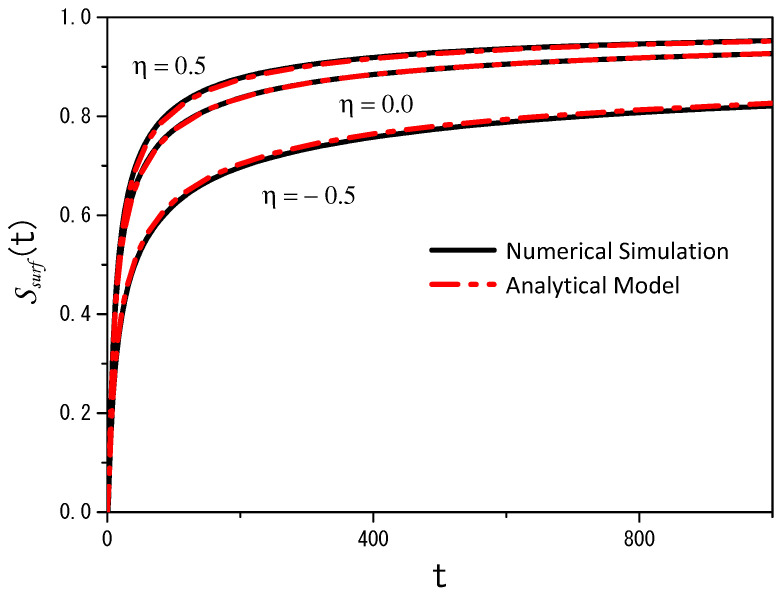
Trends of the survival probability for the adsorption–desorption case for different values of η. As described in this section, the black solid lines correspond to the numerical simulations using the Langevin equation, and the red dashed–dotted lines correspond to the analytical model. For the analytic model, we consider, with illustrative purposes, $k_{\mathrm{desor}}=0.0$, $k_{\mathrm{ads}}\to \infty$ (total absorption process), $D_{b,\|}=D_{b,⊥}=2.0$, and $z^{\prime }=4.0$, in arbitrary units. In the numerical simulations, we considered 200 k particles with the same starting conditions as in the analytical model; the desorption process is absent (rh=0%/step), D=2.0, and h=0.01.

Figure 3 illustrates an important analytical result, the survival probability, which can be constructed from the solution that accounts for the particles absorbed by the surface. Indeed, from Equation (29), we obtain the survival probability, i.e.,

(30) $$\begin{array}{c} S_{\mathrm{bulk}}\left(t\right)=\int dr_{\|}\int_{0}^{\infty }dz\rho_{\mathrm{bulk}}\left(r_{\|},z,t\right), \end{array}$$

and evaluate

(31) $$\begin{array}{c} S_{\mathrm{surf}}\left(t\right)=\int dr_{\|}\rho_{\mathrm{surf}}\left(r_{\|},t\right)=1-S_{\mathrm{bulk}}\left(t\right)\;. \end{array}$$

Both equations are related to the probability of finding particles in bulk ($S_{\mathrm{bulk}}\left(t\right)$) or on the surface ($S_{\mathrm{surf}}\left(t\right)$) and, consequently, correspond to the fractions of particles present in bulk and on the surface.

In Figure 3, we also show the result obtained by numerical simulation using the Langevin equation for the particles on the surface and in the bulk. They are defined as follows for the x−y direction:

(32) $$\begin{array}{c} \begin{array}{cc} x_{t+h}=x_{t}+\sqrt{Dh}\zeta_{x}\left(t\right), & -\infty <x<\infty \\ y_{t+h}=y_{t}+\sqrt{Dh}\zeta_{y}\left(t\right), & -\infty <y<\infty \;, \end{array} \end{array}$$

and, consequently, we have information on $r_{\|}=x\left(t\right)\hat{x}+y\left(t\right)\hat{y}$. Note that the Langevin equations for the *x* and *y* directions were used to simulate the particles on the surface and in bulk. For the *z* direction, the Langevin equation for the particles, in bulk, is given by

(33) $$\begin{array}{c} z_{t+h}=z_{t}-\left[\left(\frac{\eta Dh}{2}sign\left(z_{t}\right)\left(\right|z_{t}{\left|\right)}^{-\eta -1}\right)-(\sqrt{Dh}\left(\right|z_{t}{\left|\right)}^{-\frac{\eta }{2}})\zeta_{z}\left(t\right)\right],\;\;\;0\leq z<\infty \;. \end{array}$$

**Figure 4:**
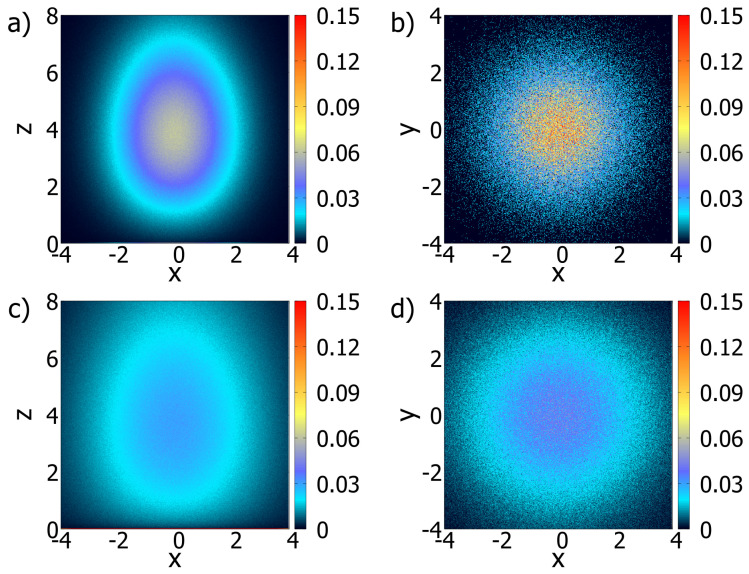
Probability density maps for simulations with η=−0.5. (**a**,**b**) represent the (x,z) and (x,y) of the bulk and surface densities at a time equal to 1.0. (**c**,**d**) correspond to (**a**,**b**) at the time 2.5. Note that the spatial dependence of the diffusion coefficient, as in Figure 1a,b directly influences the system’s behavior. This was a short run with 250 steps and 50M particles with the initial position on $\left(x_{0}=0,y_{0}=0,z_{0}=4.0\right)$. In this simulation, h=0.01, D=2.0, and the desorption process is absent.

**Figure 5:**
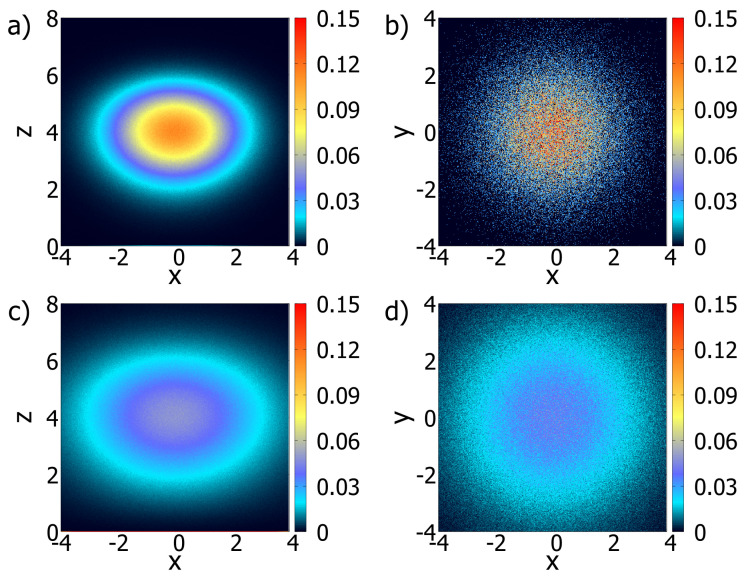
Probability density maps for simulations with η=0.5. (**a**,**b**) represent the (x,z) and (x,y) projections of the bulk and surface densities at a time equal to 1.0. (**c**,**d**) advance the time to 2.5. Apart from the value of η, all coefficients were the same as in Figure 4 and the desorption process is absent.

In these stochastic equations, $\zeta_{i}\left(t\right)$ (i=x,y, and *z*) is white Gaussian noise with a normalized deviation generated using the Box–Muller method [42]. In addition, $\langle \zeta_{i}\left(t\right)\rangle =0$, $\langle \zeta_{i}\left(t\right)\zeta_{j}\left(t^{\prime }\right)\rangle =0$ for i≠j and $\langle \zeta_{i}\left(t\right)\zeta_{i}\left(t^{\prime }\right)\rangle \propto \delta \left(t-t^{\prime }\right)$. The pseudorandom number generator utilized in this work was the maximally equidistributed combined Tausworthe generator [43] implemented via the tauss88 function from the C++ library Boost [44] (for more details, see Appendix A). For this case, we consider that the surface only absorbs particles governed by the Langevin equations, i.e., Equations (32) and (33). Figure 4 and Figure 5 illustrate the results obtained by using the previous Langevin equations for the directions *x*, *y*, and *z* when η=−0.5 and η=0.5. These figures correspond to the projections (x,z) and (x,y) to show how heterogeneity influences the dynamics aspects of the system. An animated version of the (x,z) projections can be seen at https://youtu.be/lF6WpIQI-c4 (accessed on 6 March 2024).

The second case is characterized by a surface that can adsorb and desorb at some rates, i.e., ${\hat{k}}_{\mathrm{total}}\left(s\right)=k_{\mathrm{desor}}$, ${\hat{k}}_{r}\left(s\right)=0$, and ${\hat{k}}_{\mathrm{ads}}\left(s\right)=k_{\mathrm{ads}}$. In this case, for the particles initially in bulk, from Equations (22) and (23), we obtain

(34) $$\begin{array}{c} \begin{array}{cc} {\hat{\bar{\rho }}}_{\mathrm{surf}}\left(k_{\|},s\right) & =-\frac{k_{\mathrm{ads}}}{s+D_{s}{\left|k_{\|}\right|}^{2}+k_{desorb}-sk_{\mathrm{ads}}\hat{\bar{G}}\left(0,0,z^{\prime },s\right)}\\ & \times \int_{0}^{\infty }dz^{\prime }{\bar{\varphi }}_{\mathrm{bulk}}\left(k_{\|},z^{\prime }\right)\hat{\bar{G}}\left(k_{\|},0,z^{\prime },s\right)\;, \end{array} \end{array}$$

and

(35) $$\begin{array}{c} \begin{array}{cc} {\hat{\bar{\rho }}}_{\mathrm{bulk}} & \left(k_{\|},z,s\right)=-\int_{0}^{\infty }dz^{\prime }{\tilde{\varphi }}_{\mathrm{bulk}}\left(k_{\|},z^{\prime }\right)\hat{\bar{G}}\left(k_{\|},z,z^{\prime },s\right)\\ & -\frac{sk_{\mathrm{ads}}\hat{\bar{G}}\left(k_{\|},0,z,s\right)}{s+D_{s}{\left|k_{\|}\right|}^{2}+k_{\mathrm{desor}}-sk_{\mathrm{ads}}\hat{\bar{G}}\left(k_{\|},0,0,s\right)}\int_{0}^{\infty }dz^{\prime }{\bar{\varphi }}_{\mathrm{bulk}}\left(k_{\|},z^{\prime }\right)\hat{\bar{G}}\left(k_{\|},0,z^{\prime },s\right)\;. \end{array} \end{array}$$

After obtaining the inverses of the Fourier and Laplace transforms, we obtain the following analytical results:

(36) $$\begin{array}{c} \begin{array}{cc} \rho_{\mathrm{surf}}\left(r_{\|},s\right) & =-k_{\mathrm{ads}}\int dr_{\|}^{\prime }\int_{0}^{t}dt^{\prime }G^{\left(2\right)}\left(r_{\|}-r_{\|}^{\prime },t-t^{\prime }\right)\\ & \times \int dr_{\|}^{\prime \prime }\int_{0}^{\infty }dz^{\prime }\varphi_{\mathrm{bulk}}\left(r_{\|}^{\prime }-r_{\|}^{\prime \prime },z^{\prime }\right)G\left(r_{\|},0,z^{\prime },t^{\prime }\right) \end{array} \end{array}$$

with

(37) $$\begin{array}{c} \begin{array}{cc} G^{\left(2\right)}\left(r_{\|},t\right) & =\sum_{m=0}^{n}\left(\begin{array}{c} n\\ m \end{array}\right)\int_{0}^{t}dt^{\prime }\frac{{\left(t-t^{\prime }\right)}^{\frac{1+\eta }{2+\eta }n-1}t^{\prime m}}{\Gamma \left(\frac{1+\eta }{2+\eta }n\right)\Gamma \left(1+m\right)}\\ & \times \int dr_{\|}^{\prime }e^{-k_{\mathrm{desor}}\left(t-t^{\prime }\right)}G_{D_{s},xy}\left(r_{\|}^{\prime },t-t^{\prime }\right)\frac{\partial^{m}}{\partial t^{\prime m}}[e^{-k_{\mathrm{desor}}t^{\prime }}G_{D_{b,\|},xy}\left(r_{\|}-r_{\|}^{\prime },t^{\prime }\right)], \end{array} \end{array}$$

and

(38) $$\begin{array}{ccc} \rho_{\mathrm{bulk}}\left(r_{\|},z,s\right) & = & -\int dr_{\|}^{\prime }\int_{0}^{\infty }dz^{\prime }\varphi_{\mathrm{bulk}}\left(r_{\|}^{\prime },z^{\prime }\right)G\left(r_{\|}-r_{\|}^{\prime },z,z^{\prime },t\right)\\ & - & k_{\mathrm{ads}}\int dr_{\|}^{\prime }\int_{0}^{t}dt^{\prime }G^{\left(2\right)}\left(r_{\|}-r_{\|}^{\prime },t-t^{\prime }\right)\int_{0}^{t^{\prime }}dt^{\prime \prime }\int dr_{\|}^{\prime \prime }G\left(r_{\|}^{\prime }-r_{\|}^{\prime \prime },0,z,t^{\prime }-t^{\prime \prime }\right)\\ & \times & \int dr_{\|}^{\prime \prime \prime }\int_{0}^{\infty }dz^{\prime }\varphi_{\mathrm{bulk}}\left(r_{\|}^{\prime \prime }-r_{\|}^{\prime \prime \prime },z^{\prime }\right)G\left(r_{\|}^{\prime \prime \prime },0,z^{\prime },t^{\prime \prime }\right)\;. \end{array}$$

**Figure 6:**
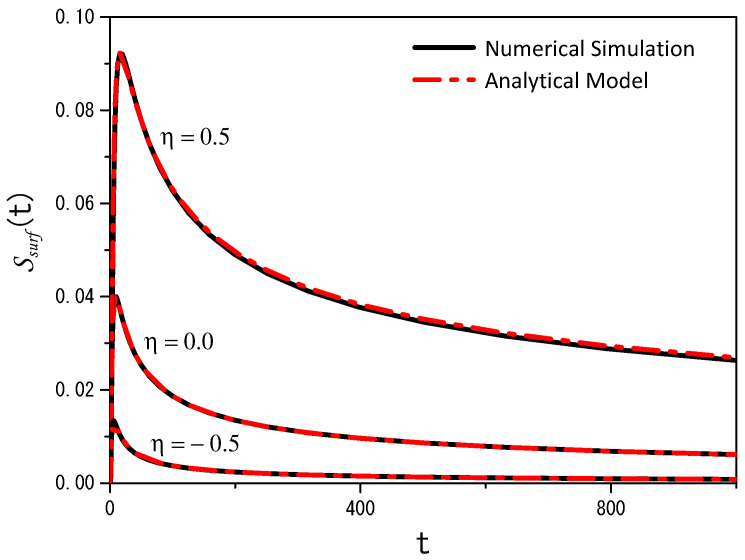
Trends of the survival probability for the adsorption–desorption case with different values of η. The black solid lines correspond to the numerical simulations, as described in the text, whereas the red dashed–dotted lines correspond to the analytical model. We have used the model to adjust the data obtained from the numerical simulations with the Langevin equations. Thus, the kinetic parameters, i.e., $k_{\mathrm{ads}}$ and $k_{\mathrm{desor}}$, adjusted both the model and the numerical simulations. The parameter values for η=0.5 were $k_{\mathrm{desor}}=1.5$ and $k_{\mathrm{ads}}=0.9$; for η=0.0 were $k_{\mathrm{desor}}=0.45$ and $k_{\mathrm{ads}}=2.9$; and for η=−0.5 were $k_{\mathrm{desor}}=0.2$ and $k_{\mathrm{ads}}=4.5$. For simplicity, we consider $D_{b,\|}=D_{b,⊥}=2.0$ and $z^{\prime }=4.0$, in arbitrary units. These numerical simulations utilized the parameters shown in Figure 3, except for rh, which is equal to 5%/step.

In Figure 6, the number of particles adsorbed, obtained from the analytical and numerical points of view, is represented. We underline that the numerical simulations were performed considering that the desorption process of the particles, from the surface to the bulk, follows the conditions

(39) $$\begin{array}{c} \begin{array}{cc} z_{t+h}=0.5 & —\;\mathrm{desorption}\;\mathrm{probability}\;(\mathrm{surface}\;\mathrm{to}\;\mathrm{the}\;\mathrm{bulk})\;—\;p_{des}=rh\\ z_{t+h}=z_{t}=0 & —\;\mathrm{non-desorption}\;\mathrm{probability}\;—\;p_{stay}=1-rh \end{array}\; \end{array}$$

(for more details, see Appendix A).

## 3. Discussion and Conclusions

This work investigates a diffusion process in a heterogeneous medium with adsorption–desorption occurring on a surface. The particles adsorbed can diffuse on the surface and may desorb back into the bulk after some time. The bulk process is governed by a diffusion equation with a spatially dependent diffusion coefficient. The surface adsorption–desorption process is described by the kinetic functions $k_{\mathrm{ads}}\left(t\right)$ and $k_{\mathrm{desor}}\left(t\right)$, which represent the kernels in Equation (3).

We performed numerical simulations using the Langevin equation with multiplicative noise to gain additional insight into the diffusion process from a different perspective. This approach complements the analytical model. Figure 3 and Figure 6 compare the analytical and numerical results, showing good agreement for the cases analyzed. The first case was considered to have a purely adsorbing surface, which means that desorption was absent and all particles were adsorbed. The second case incorporated desorption, allowing particles to return to the bulk after some time. For this case, we adjusted the parameters related to adsorption–desorption in the analytical model to match the numerical results.

These findings extend previous work in [45,46] by incorporating bulk inhomogeneity and surface diffusion. We believe the results presented here can contribute to the study of diffusion processes with surface adsorption–desorption and the possibility of surface diffusion. 

## Figures and Tables

**Figure A1:**
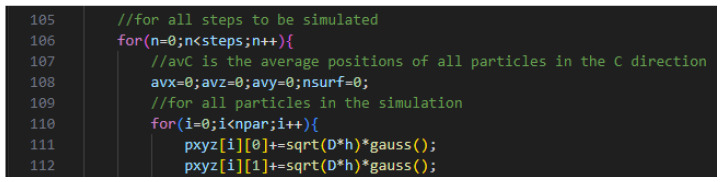
pxyz represents the position matrix. The first index is the *i*-th — particle, and the second is the direction modulus. It is important to emphasize that the calculation of diffusion in both the x and y directions is always performed. The function “gauss ( )” generates the Gaussian noise, which will be shown at the end of the Appendix A.

**Figure A2:**
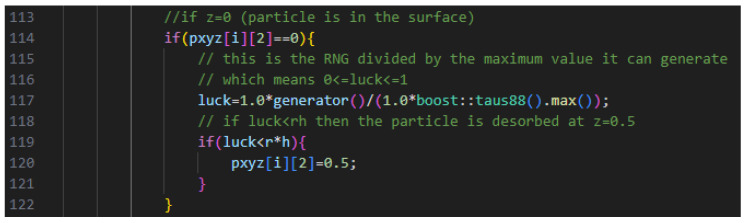
This part of the code applies Equation (A2) to model stochastic desorption. RNG means random number generator.

**Figure A3:**
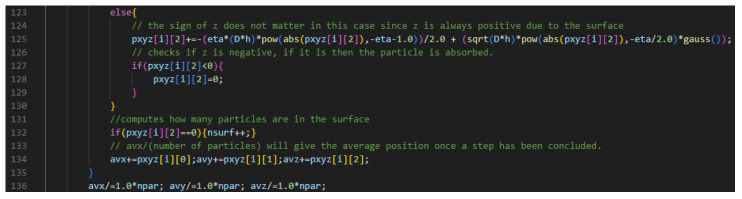
Here, we calculated diffusion in z and checked for adsorption. Then, “nsurf” can be used to calculate the ratio of particles on the surface.

**Figure A4:**
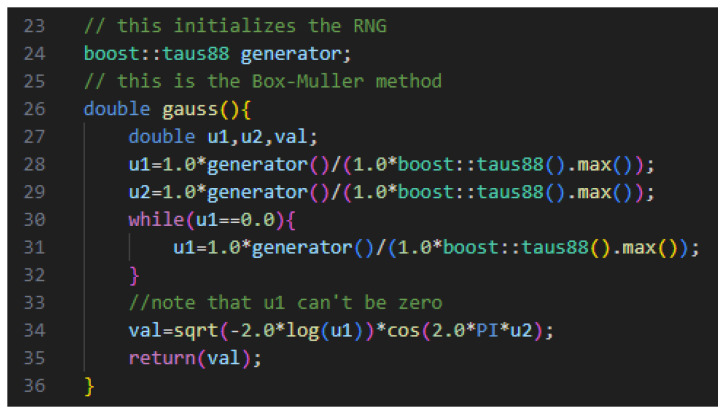
This code initializes the RNG and applies the Box–Muller method. Note that 0<u1≤1 and 0≤u2≤1.

## Data Availability

The data presented in this study are available on request from the corresponding author.

## Appendix A. Numerical Approach

Let us describe, in more detail, the numerical procedure to simulate the first and second scenarios handled in Section 2. We will begin by presenting the Langevin equations that govern the dynamics of the particles in the system. They are

(A1) $$\begin{array}{c} \begin{array}{cc} x_{t+h}=x_{t}+\sqrt{Dh}\zeta_{x}\left(t\right), & -\infty <x<\infty \;,\\ y_{t+h}=y_{t}+\sqrt{Dh}\zeta_{y}\left(t\right), & -\infty <y<\infty \;,\\ z_{t+h}=z_{t}-((\frac{\eta Dh}{2}sign\left(z_{t}\right)\left(\right|z_{t}{\left|\right)}^{-\eta -1})-(\sqrt{Dh}\left(\right|z_{t}{\left|\right)}^{-\frac{\eta }{2}})\zeta_{z}\left(t\right)) & \;\;\;\;0\leq z<\infty \;. \end{array} \end{array}$$

For simplicity, we consider the same diffusion coefficient for the surface and bulk. We assume that the desorption process of the particles on the surface follows these stochastic equations:

(A2) $$\begin{array}{c} \begin{array}{cc} z_{t+h}=0.5 & \;—\;\mathrm{desorption}\;\mathrm{probability}\;(\mathrm{surface}\;\mathrm{to}\;\mathrm{the}\;\mathrm{bulk})\;—\;p_{des}=rh\\ z_{t+h}=z_{t}=0 & \;—\;\mathrm{non-desorption}\;\mathrm{probability}\;—\;p_{stay}=1-rh. \end{array}\; \end{array}$$

Once the system’s dynamics are given, we can start with the C++ code. The setup is as follows.

If the particle is on the surface, we check for desorption.

If the particle is not on the surface, it is in the bulk. The code will compute the diffusion in z and check if the particle was absorbed.

Finally, we can generate the Gaussian distribution.

## References

[1] Hoda N., Kumar S. (2008). Brownian dynamics simulations of polyelectrolyte adsorption in shear flow: Effects of solvent quality and charge patterning. J. Chem. Phys..

[2] Egan M., Akdeniz B.C., Tang B.Q. (2022). Stochastic reaction and diffusion systems in molecular communications: Recent results and open problems. Digit. Signal Process..

[3] Gardiner C.W. (1996). Handbook of Stochastic Methods.

[4] Metzler R., Klafter J. (2000). The random walk’s guide to anomalous diffusion: A fractional dynamics approach. Phys. Rep..

[5] Evangelista L.R., Lenzi E.K. (2018). Fractional Diffusion Equations and Anomalous Diffusion.

[6] Metzler R. (2019). Brownian motion and beyond: First-passage, power spectrum, non-Gaussianity, and anomalous diffusion. J. Stat. Mech. Theory Exp..

[7] Thurber G.M., Schmidt M.M., Wittrup K.D. (2008). Factors determining antibody distribution in tumors. Trends Pharmacol. Sci..

[8] Bisquert J., Compte A. (2001). Theory of the electrochemical impedance of anomalous diffusion. J. Electroanal. Chem..

[9] Niu Q., Wang D. (2019). Probing the polymer anomalous dynamics at solid/liquid interfaces at the single-molecule level. Curr. Opin. Colloid Interface Sci..

[10] Woringer M., Izeddin I., Favard C., Berry H. (2020). Anomalous Subdiffusion in Living Cells: Bridging the Gap Between Experiments and Realistic Models Through Collaborative Challenges. Front. Phys..

[11] Di Pierro M., Potoyan D.A., Wolynes P.G., Onuchic J.N. (2018). Anomalous diffusion, spatial coherence, and viscoelasticity from the energy landscape of human chromosomes. Proc. Natl. Acad. Sci. USA.

[12] Burnecki K., Kepten E., Garini Y., Sikora G., Weron A. (2015). Estimating the anomalous diffusion exponent for single particle tracking data with measurement errors - An alternative approach. Sci. Rep..

[13] Ledesma-Duraán A., Hernández S., Santamaría-Holek I. (2017). Effect of Surface Diffusion on Adsorption–Desorption and Catalytic Kinetics in Irregular Pores. I. Local Kinetics. J. Phys. Chem. C.

[14] Ledesma-Durán A., Hernández S.I., Santamaría-Holek I. (2017). Effect of Surface Diffusion on Adsorption–Desorption and Catalytic Kinetics in Irregular Pores. II. Macro-Kinetics. J. Phys. Chem. C.

[15] Campagnola G., Nepal K., Schroder B.W., Peersen O.B., Krapf D. (2015). Superdiffusive motion of membrane-targeting C2 domains. Sci. Rep..

[16] Chipot C., Comer J. (2016). Subdiffusion in Membrane Permeation of Small Molecules. Sci. Rep..

[17] Longeville S., Stingaciu L.R. (2017). Hemoglobin diffusion and the dynamics of oxygen capture by red blood cells. Sci. Rep..

[18] Jacobson K., Liu P., Lagerholm B.C. (2019). The Lateral Organization and Mobility of Plasma Membrane Components. Cell.

[19] Ramadurai S., Holt A., Krasnikov V., van den Bogaart G., Killian J.A., Poolman B. (2009). Lateral Diffusion of Membrane Proteins. J. Am. Chem. Soc..

[20] Renner M., Domanov Y., Sandrin F., Izeddin I., Bassereau P., Triller A. (2011). Lateral Diffusion on Tubular Membranes: Quantification of Measurements Bias. PLoS ONE.

[21] Kim S.G., Wang S.H., Ok C.M., Jeong S.Y., Lee H.S. (2017). Lateral diffusion of graphene oxides in water and the size effect on the orientation of dispersions and electrical conductivity. Carbon.

[22] Hu C., Wang X., Song B. (2020). High-performance position-sensitive detector based on the lateral photoelectrical effect of two-dimensional materials. Light. Sci. Appl..

[23] Metzler R., Glöckle W.G., Nonnenmacher T.F. (1994). Fractional model equation for anomalous diffusion. Physica A.

[24] O’Shaughnessy B., Procaccia I. (1985). Analytical Solutions for Diffusion on Fractal Objects. Phys. Rev. Lett..

[25] Dekeyser R., Maritan A., Stella A.L. (1994). Diffusion on fractal substrates. Diffusion Processes: Experiment, Theory, Simulations, Proceedings of the Vth Max Born Symposium, Kudowa, Poland, 1–4 June 1994.

[26] Boffetta G., Sokolov I.M. (2002). Relative Dispersion in Fully Developed Turbulence: The Richardson’s Law and Intermittency Corrections. Phys. Rev. Lett..

[27] Su N., Sander G., Liu F., Anh V., Barry D. (2005). Similarity solutions for solute transport in fractal porous media using a time- and scale-dependent dispersivity. App. Math. Model..

[28] Anderson A.N., Crawford J.W., McBratney A.B. (2000). On diffusion in fractal soil structures. Soil Sci. Soc. Am. J..

[29] Brault P., Josserand C., Bauchire J.M., Caillard A., Charles C., Boswell R.W. (2009). Anomalous Diffusion Mediated by Atom Deposition into a Porous Substrate. Phys. Rev. Lett..

[30] Gervais T., Jensen K.F. (2006). Mass transport and surface reactions in microfluidic systems. Chem. Eng. Sci..

[31] Roshandel R., Ahmadi F. (2013). Effects of catalyst loading gradient in catalyst layers on performance of polymer electrolyte membrane fuel cells. Renew. Energy.

[32] Nazeeruddin M.K., Baranoff E., Grätzel M. (2011). Dye-sensitized solar cells: A brief overview. Sol. Energy.

[33] Zola R.S., Lenzi E.K., Evangelista L.R., Barbero G. (2007). Memory effect in the adsorption phenomena of neutral particles. Phys. Rev. E.

[34] Fogler H.S. (2010). Essentials of Chemical Reaction Engineering.

[35] Crank J. (1979). The Mathematics of Diffusion.

[36] Arfken G., Weber H., Harris F. (2013). Mathematical Methods for Physicists: A Comprehensive Guide.

[37] Wyld H.W. (1999). Mathematical Methods for Physics.

[38] Ali I., Kalla S. (1999). A generalized Hankel transform and its use for solving certain partial differential equations. ANZIAM J..

[39] Garg M., Rao A., Kalla S.L. (2007). On a generalized finite Hankel transform. Appl. Math. Comput..

[40] Nakhi Y.B., Kalla S.L. (2003). Some boundary value problems of temperature fields in oil strata. Appl. Math. Comput..

[41] Xie K., Wang Y., Wang K., Cai X. (2010). Application of Hankel transforms to boundary value problems of water flow due to a circular source. Appl. Math. Comput..

[42] Scott D.W. (2011). Box–Muller transformation. WIREs Comput. Stat..

[43] L’Ecuyer P. (1996). Maximally Equidistributed Combined Tausworthe Generators. Math. Comput..

[44] https://www.boost.org/.

[45] Ndiaye P., Tavares F., Lenzi E., Evangelista L., Ribeiro H., Marin D., Guilherme L., Zola R. (2021). Sorption–desorption, surface diffusion, and memory effects in a 3D system. J. Stat. Mech. Theory Exp..

[46] Koltun A.P.S., Lenzi E.K., Lenzi M.K., Zola R.S. (2021). Diffusion in Heterogenous Media and Sorption—Desorption Processes. Fractal Fract..

